# Supplementary material for: Comparative multiomics analysis of cell physiological state after culture in a basket bioreactor
Source: Sci Rep. 2022 Nov 23;12:20161. doi: 10.1038/s41598-022-24687-4 (PMC9686226; doi:10.1038/s41598-022-24687-4)
Supplement: Supplementary file 1 — Supplementary Information 1. [file 41598_2022_24687_MOESM1_ESM.zip › raw data/Transcriptomic raw data/6.Enrichment/2.KEGG/TE1vsTC1/ALL/TE1vsTC1.all_KEGGdot.pdf]

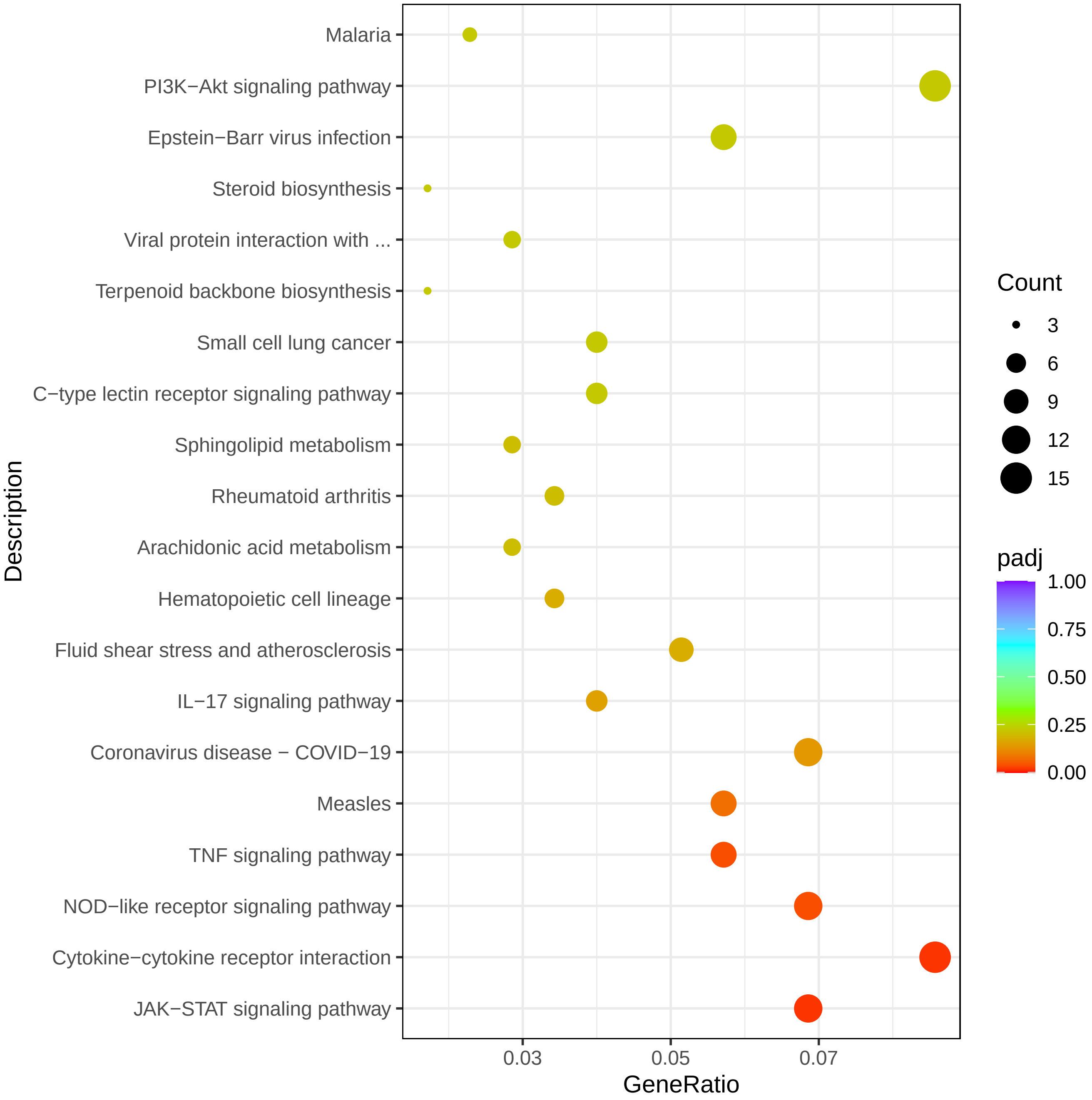

Malaria
PI3K−Akt signaling pathway
Epstein−Barr virus infection
Steroid biosynthesis
Viral protein interaction with ...
Terpenoid backbone biosynthesis
Small cell lung cancer
C−type lectin receptor signaling pathway
Sphingolipid metabolism
Rheumatoid arthritis
Arachidonic acid metabolism
Hematopoietic cell lineage
Fluid shear stress and atherosclerosis
IL−17 signaling pathway
Coronavirus disease − COVID−19
Measles
TNF signaling pathway
NOD−like receptor signaling pathway
Cytokine−cytokine receptor interaction
JAK−STAT signaling pathway
Description
0.03
0.05
0.07
GeneRatio
Count
3
6
9
12
15
padj
1.00
0.75
0.50
0.25
0.00
